# Estimating model error covariances using particle filters

**DOI:** 10.1002/qj.3132

**Published:** 2017-11-08

**Authors:** Mengbin Zhu, Peter J. van Leeuwen, Weimin Zhang

**Affiliations:** ^1^ Academy of Ocean Science and Engineering, National University of Defense Technology Changsha China; ^2^ Department of Meteorology University of Reading UK; ^3^ National Centre for Earth Observation, University of Reading UK

**Keywords:** model error covariance, non‐degeneracy, particle filter, localization

## Abstract

A method is presented for estimating the error covariance of the errors in the model equations in observation space. Estimating model errors in this systematic way opens up the possibility to use data assimilation for systematic model improvement at the level of the model equations, which would be a huge step forward. This model error covariance is perhaps the hardest covariance matrix to estimate. It represents how the missing physics and errors in parametrizations manifest themselves at the scales the model can resolve.

A new element is that we use an efficient particle filter to avoid the need to estimate the error covariance of the state as well, which most other data assimilation methods do require. Starting from a reasonable first estimate, the method generates new estimates iteratively during the data assimilation run, and the method is shown to converge to the correct model error matrix. We also investigate the influence of the accuracy of the observation error covariance on the estimation of the model error covariance and show that, when the observation errors are known, the model error covariance can be estimated well, but, as expected and perhaps unavoidably, the diagonal elements are estimated too low when the observation errors are estimated too high, and vice versa.

## 1 INTRODUCTION

Linear and linearized data assimilation (DA) rely on prescribing or accurately estimating covariance matrices, related to the (near‐)Gaussian assumptions on the probability densities in Bayes Theorem. For example, standard 3D‐ and 4D‐Var stand or fall with an accurately prescribed state covariance **B**, and an accurately prescribed observation error covariance **R**. Especially for the former, numerous person‐years are typically needed to tune the prior state covariance matrix. Ensemble Kalman Filters and Smoothers need an accurate **R**, and their prior covariance at each observation time is the sample covariance from the ensemble (Evensen, 1994; Evensen and van Leeuwen, 2000). Because of the small ensemble size that can be afforded in numerical weather prediction, that matrix is rank deficient, underestimates the covariances, and has large Monte Carlo errors, resulting in spurious long‐range correlations. *A* *d* *h* *o* *c* methods like inflation and localization are needed to generate useful prior covariances from the sample covariance matrix (Anderson, 2007; 2009). (Of course, for high‐dimensional systems the covariance is never calculated explicitly, but the issues and partial solutions mentioned above are still relevant.) Several issues arise when using inflation and localization, for instance how to localize for an observation that is an integral along a line. In so‐called hybrid methods that combine variational and Ensemble Kalman Filter methods, the estimation problems do not disappear, and in iterative ensemble smoothers like 4DEnsVar one has to estimate space–time covariances from an ensemble, leading to further problems with localization in time (Liu *et al.*, 2014; Buehner *et al.*, 2010).

Several methods to estimate state covariances have been developed, and Bannister (2008a; 2008b) gives a good overview of what has been done. The so‐called Desroziers diagnostic (Desroziers *et al.*, 2005) has become very popular because of its relative simplicity. It uses the statistics of innovations and analysis minus observations to estimate either the prior covariance **B** in observation space, or the observation error covariance **R**, or sometimes both, exploring relations from linear estimation theory. Estimation of **R** is very popular nowadays because it has become clear that correlations between observation errors need to be taken into account to extract most information from the observations and to obtain the best analysis (Stewart *et al.*, 2013; Weston *et al.*, 2014). Another issue is that the error covariances **B** and **R** should have different characteristics, such as length‐scales, to be able to estimate both matrices together.

Recently the Desroziers method has also been used to estimate the model error covariance **Q**. Todling (2008; 2013) gives an alternative method to diagnose model error, which requires two overlapping DA systems, one sequential filter and one fixed lag‐1 smoother. The model error diagnosed by Todling (2008; 2013) comes from the differences between two model states. Todling stresses the problem that estimating **Q** while **R** and **B** are not known precisely can lead to attributing structures to **Q** that belong in **R** and especially **B**, and vice versa.

A major advantage of particle filters is that the prior state error covariance does not play a role, so **B** does not have to be prescribed or estimated. So, under the perfect model assumption, the only matrix to be estimated is **R**. No studies have been performed in this direction to our knowledge. One of the reasons is that standard particle filters are degenerate when the number of observations is large, meaning that the weight that each particle obtains during the assimilation varies enormously over the particles, with one particle having a much larger weight than all the others. This then means that the ensemble effectively has collapsed onto that one particle.

Three solutions have been presented in the literature: localized particle filters, like the Local Ensemble Transform Particle Filter by Reich (1995), and the localized particle filter by Poterjoy (1986), methods that combine Particle Filters with Ensemble Kalman Filters (Lei and Bickel, 2010; Frei and Künsch, 2013), and methods that explore the proposal density freedom (Chorin and Tu, 2009; van Leeuwen, 2015a; Zhu *et al.*, 2016). Reich (1995) estimates a local transformation matrix via optimal transportation to transform the weighted prior ensemble into an equal‐weighted posterior ensemble, while Poterjoy (1986) employs a very delicate smoothing procedure to ensure smooth resampled particles. A problem with particle filters that use localization is that the localization radius has to be taken very small to avoid this weight collapse, as the variance of the weights scales with the number of independent observations within the localization area. The Poterjoy (1986) scheme avoids this issue by setting a minimal weight for each particle, and this issue has not been discussed yet for the other schemes. Furthermore, issues with localization as mentioned above for ensemble Kalman filters play a role here too, and also inflation has to be applied. The second method needs to estimate sample covariance matrices for the EnKF part of the algorithm, again with the same issues as above. The third method needs model errors to be able to move the particles in state space different from the deterministic model equations. Hence for particle filters practical for high‐dimensional geophysical problems, we need localization or we need to estimate **Q**.

Interestingly, there is a huge advantage having to estimate **Q** over **B**. **B** is just a statistical quantity that tells us something about the accuracy of a best estimate, while **Q** contains information about how and where our model is wrong. So estimating **Q** gives us a direct route to improving our model, one of the holy grails of DA, in which very little progress has been made in the last 30 years.

This article is organized as follows. The new scheme for estimating the model error covariance is described in section 2; other variants are described in Appendix 4, and details on convergence are presented in Appendix A2. The method is tested on the 1000‐dimensional Lorenz‐96 model and discussed further in section 3. A summary and conclusions are included in section 4.

## 2 ESTIMATING THE MODEL ERROR COVARIANCE

In the Desroziers diagnostic, one calculates the innovation and/or the analysis minus observation statistics, or the statistics of the combination of these two. In a particle filter there is no unique best forecast or analysis. One could choose the ensemble mean, but that is not always the natural choice, for instance the probability density functions (pdfs) can be bimodal with modes of similar magnitude. So, instead, it is natural to explore the innovations of all particles. The model equations are denoted as

(1) $$x^{n}=f(x^{n-1})+\beta^{n},$$

in which the superscript denotes the time index, *f*(..) denotes the nonlinear deterministic model, and *β* ^*n*^ denotes the stochastic term representing the missing physics in the model, taken as additive noise drawn from *N*(0,**Q**).

To simplify the notation we introduce $f_{i}=f(x_{i}^{n-1})$ with *i* the particle index, $\bar{f}$ is the ensemble mean of the *f*_*i*_, and $f_{t}=f(x_{t}^{n-1})$ in which the subscript t denotes the true state. We then find

(2) $$\begin{array}{ll} y^{n}- & H(f_{i})\;\\ & =y^{n}-H(x_{t}^{n})+H(x_{t}^{n})-H(f_{i})\;\\ & ={ɛ}_{o}^{n}+H(x_{t}-f_{t}+f_{t})-H(f_{i})\;\\ & \approx {ɛ}_{o}^{n}+\tilde{H}(x_{t}-f_{t})+H(f_{t})-H(f_{i})\;\\ & ={ɛ}_{o}^{n}+\tilde{H}{ɛ}_{q}^{n}+H(f_{t})-H(\bar{f})+H(\bar{f})-H(f_{i}), \end{array}$$

in which ${ɛ}_{o}^{n}=y^{n}\;-\;H(x_{t}^{n})$, ${ɛ}_{q}^{n}=x_{t}\;-\;f_{t}$. This allows us to write

(3) $$y^{n}-H(f_{i})\approx {ɛ}_{o}^{n}+\tilde{H}{ɛ}_{q}^{n}+v_{t}^{n}+v_{i}^{n},$$

in which the approximation is due to the Taylor series expansion of **H** at point *f*_*t*_ and $\tilde{H}$ is the derivative of **H** to its argument at that point. If **H** is linear, the above is true without approximation taking $\tilde{H}=H$.

From now on we will denote *H*(..) (note the parentheses) as the full nonlinear *H*, and *H*.. (without parentheses) for the derivative of *H*(..) at the true value. Furthermore,

(4) $$v_{i}^{n}=H(\bar{f})-H(f_{i})$$

and similarly for $v_{t}^{n}=H(f_{t})-H(\bar{f})$.

Assuming all terms in the particle innovations are independent, we can now form

(5) $$\begin{array}{ll} C & =\frac{1}{N-1}\sum_{i}\{y^{n}-H(f_{i})\}{\left\{y^{n}-H(f_{i})\right\}}^{T}\;\\ & =R+{HQH}^{T}+\frac{1}{N-1}\sum_{i}v_{t}^{n}{v_{t}^{n}}^{T}+\frac{1}{N-1}\sum_{i}v_{i}^{n}{v_{i}^{n}}^{T}, \end{array}$$

in which we use the linearized **H** in the last line exploring the notation explained above. We now note that, for a well‐performing DA system, on average the distance of an ensemble member to the ensemble mean is equal to the distance between the truth and the ensemble mean, so the ensemble variance is equal to the square of the RMSE, so that the last two terms are approximately equal. This allows us to approximate

(6) $$C=R+{HQH}^{T}+2V$$

with

(7) $$\begin{array}{ll} V & =\frac{1}{N-1}\sum_{i}v_{i}^{n}{v_{i}^{N}}^{T}\;\\ & =\frac{1}{N-1}\sum_{i}\{H(\bar{f})-H(f_{i})\}{\left\{H(\bar{f})-H(f_{i})\right\}}^{T}.\; \end{array}$$

This then allows us to find an estimate for **H** **Q** **H** ^*T*^ as:

(8) $${HQH}^{T}=C-R-2V.$$

As with the Desroziers diagnostics, we could also try to use the statistics of $\left(y^{n}-Hx_{i}^{n}\right)$, i.e. the difference between observation at time *n* and the ensemble members at time *n*. In the appendix we show that, at least for the particle filter we use here, these methods do not reveal new information, but they rather complicate the computation of **H** **Q** **H** ^*T*^.

The ensemble estimate of *C* and *V*, and hence **H** **Q** **H** ^*T*^ will be of very low rank because of the limited ensemble size, typically of order 10 to 250. This leads to a large sampling error. To this end, we apply averaging over both space and time of the estimated **H** **Q** **H** ^*T*^, as described below.

An important point is that the scheme described above only updates **Q** in observation space, so **HQH** ^T^, and the rest of **Q** is untouched. This is a shortcoming of the estimation scheme, similar to estimating **H** **B** **H** ^*T*^ using innovations in linear estimation theory. A partial solution is to identify parts of the state space with similar physics and copy **HQH** ^*T*^ from observed parts of the state space to unobserved parts with similar physics. For parts of the state that have no observed counterpart defined in this way we would have to rely on interpolation, with all problems attached to that. We have no full solution to this problem. In the following we assume we can estimate **Q** completely, and in the numerical experiments we assume that we observe the whole state space.

### 2.1 Temporal smoothing

The temporal averaging is described by the following algorithm.


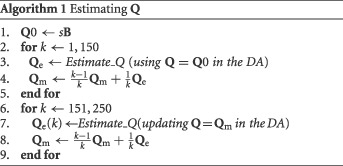


In the algorithm, **Q**_e_(*k*) is the estimated model error covariance in time step *k* and **Q**_m_ is the average of the **Q**_e_ matrices in the past *k* time steps. *s* is a scalar which is multiplied by **B** to find an initial value of **Q**0. This **B** matrix comes from the prior at the start of the DA procedure, and plays no role in the rest of the algorithm or the particle filter. If better initial estimates of **Q**0 are available, they should be used, of course. The algorithm is split in two parts. The first part uses the first‐guess model error covariance **Q**0 as the current value in the first 150 time steps of the DA. So, we do estimate **Q** at each observation step, but we do not use that raw estimate yet in the DA. The second part uses the average of the estimated model error covariances as the current estimate of model error covariance in the DA. The reason for this two‐stage algorithm is that, because of the relatively small ensemble size, the instantaneous estimates for **Q** have large sampling error, and the temporal averaging will increase this sample size. The initial averaging is taken here over 150 time steps in the pseudocode and the total running time of the whole scheme is 250 time steps, but that can (and should) be tested for each DA system separately.

### 2.2 Spatial smoothing

Because of the small ensemble size, the temporal filtering will not be enough, or has to be done over an unrealistically long time, to eliminate sampling error even in places where the correlations are high. To this end we smooth the elements over areas that we know have similar physics. In this paper two ways of smoothing are applied, localization to smooth perpendicular to the diagonal, and a simple 10 grid‐point moving average over the diagonals of the estimated covariance matrix.

Localization is a way of increasing the rank of the estimated model error covariance matrix and eliminating spurious long‐distance correlations. A simple localization strategy has been implemented here, which is the covariance localization (Houtekamer and Mitchell, 2001). Covariance localization is performed by element‐wise (Shur) multiplication of the model error covariance matrix with a predefined correlation matrix (denoted as L here) representing a decaying function of distance:

(9) $$Q_{s}^{k}=L\circ Q_{m}^{k}.$$

We use a localization radius of five grid points. Many other localization methods could be considered, tailored to specific applications.

### 2.3 Convergence of the method

In the following we discuss the convergence properties of the scheme used to estimate the model error covariance matrix **HQH** ^T^. The actual convergence of the method will depend on the particle‐filter method used. Here we provide results using the Implicit Equal‐Weights Particle Filter (IEWPF) of Zhu *et al.* (2016) as an example of how one could go about such a proof. We first describe that particle filter briefly, followed by the actual proof.

#### 2.3.1 The implicit equal‐weights particle filter

The Implicit Equal‐Weight Particle Filter (IEWPF) is described in detail in Zhu *et al.* (2016). It is a particle filter that uses a proposal density as follows. For simplicity we concentrate only on the states at time *n* and *n*−1, and assume that all observations up to time *n*−1 have been used to find a set of particles at *n*−1 with weights $w_{i}^{n-1}$. Then we can write

(10) $$\begin{array}{ll} & p(x^{n},x^{n-1}|y^{n})=\frac{p(y^{n}|x^{n})}{p(y^{n})}p(x^{n}|x^{n-1})p(x^{n-1})\;\\ & =\frac{p(y^{n}|x^{n})}{p(y^{n})}p(x^{n}|x^{n-1})\frac{1}{N}\sum_{i}\{\delta (x^{n-1}-x_{i}^{n-1})\}\;\\ & =\frac{p(y^{n}|x^{n})}{Np(y^{n})}\;\\ & \;\;\;\times \sum_{i}\frac{p(x^{n}|x_{i}^{n-1})}{q(x^{n}|X^{n-1},y^{n})}q(x^{n}|x^{n-1},y^{n})\{\delta (x^{n-1}-x_{i}^{n-1})\}\; \end{array}$$

in which $q(x^{n}|X^{n-1},y^{n})$ is the proposal density that is conditioned on all particles at time *n*−1, indicated by the $X^{n-1}$. It will be chosen such that the weights of all particles are equal. We note that, no matter what we choose for *q*, the weights will be given by

(11) $$w_{i}=\frac{p(y^{n}|x_{i}^{n})}{p(y^{n})}\frac{p(x_{i}^{n}|x_{i}^{n-1})}{q(x_{i}^{n}|X^{n-1},y^{n})}.$$

Instead of drawing directly from *q*, which will be complicated because of the equal weight procedure, we draw implicitly from a Gaussian *q*(*ξ*), leading to weights

(12) $$w_{i}=\frac{p(y^{n}|x_{i}^{n})}{p(y^{n})}\frac{p(x_{i}^{n}|x_{i}^{n-1})}{q(\xi )}\|\frac{dx}{d\xi }\|.$$

As explained in Zhu *et al.* (2016), equal‐weight particles can be constructed for instance by assuming

(13) $$x_{i}^{n}=f(x_{i}^{n-1})+K\{y^{n}-Hf(x_{i}^{n-1})\}+\alpha_{i}^{1/2}P^{1/2}\xi_{i}^{n},$$

where **K**=**Q** **H** ^*T*^(**HQH** ^*T*^+**R**)^−1^ and **P**=(**Q** ^−1^+**H** ^*T*^ **R** ^−1^ **H**)^−1^, where **Q** is the model error covariance matrix, **H** is the observation operator and **R** is the observation error covariance matrix, and in which *α*_*i*_ is a free parameter. Using this expression in the expression for the weights, the weight of particle *i* becomes a nonlinear function of parameter *α*_*i*_, for each particle index *i*. We now set a target weight as the lowest maximal weight of all particles, so

(14) $$w_{target}={min}_{i}\{{max}_{x_{i}}(w_{i})\}$$

and fix the *α*s such that the weight of each particle is equal to this target weight by solving

(15) $$w_{i}(\alpha_{i})=w_{target}.$$

As mentioned, full details are given in Zhu *et al.* (2016).

#### 2.3.2 Convergence results

The proof of convergence is rather lengthy and is provided in Appendix A2. The numerical experiments in the next section confirm this convergence on a nonlinear 1000‐dimensional problem.

## 3 EXPERIMENTS

In this section we will verify our scheme in a moderately high‐dimensional setting of the Lorenz 1996 model. The Lorenz 96 model (Lorenz, 2008) is a dynamical nonlinear model given by:

(16) $$\frac{dx_{j}}{dt}=-x_{j-2}x_{j-1}+x_{j-1}x_{j+1}-x_{j}+F,$$

where *x*_*j*_ is the state variable of the model at position *j* and *F* is a forcing constant, which is typically chosen as 8 for chaotic behaviour. The dimension of Lorenz 96 model is chosen as 1000 for all experiments discussed here. We use a time step of Δ*t*=0.05 with a fourth Runge–Kutta scheme for the deterministic part and an Euler–Maruyama scheme for the stochastic part of the model.

The detailed description of the IEWPF scheme can be found in Zhu *et al.* (2016). For the IEWPF scheme we choose the initial background‐error covariance matrix **B** as a tridiagonal matrix with main diagonal value 1 and sub‐/super‐diagonal value 0.25. The true model error covariance matrix **Q**_*t*_ is also chosen as a tridiagonal matrix with main diagonal value 0.2 and both sub‐ and super‐diagonal values are 0.05 for all the experiments in this section. The observation error covariance denoted as **R** is chosen as a diagonal matrix, with variances varying with experiment. The initial ensemble member perturbations are generated by random sampling from a Gaussian with covariance matrix the background‐error covariance and with zero mean.

To illustrate the method, we observe every grid point at every time step for the model evolution process for all the experiments in this paper. This approach was taken because it is unclear how results from extensive experimentation with different observation strategies will carry over to other models.

The typical total evolving time of the modelling scheme is set to be 250 time steps, with a few longer experiments to study convergence. The temporal averaging time is set to 150 time steps and the second iterative part of the estimation scheme is set to 100 time steps for all the experiments except for those described in section 3.4.

### 3.1 The importance of spatial smoothing

The spatial smoothing strategy is described in section 2.2, and the localization radius is chosen to be five grid points, with a ten‐grid‐point moving average along the diagonal.

We choose **R**=0.005 and the initial first guess **Q**0=0.25**B**. The number of ensemble members is set to be 40.

**Figure 1:**
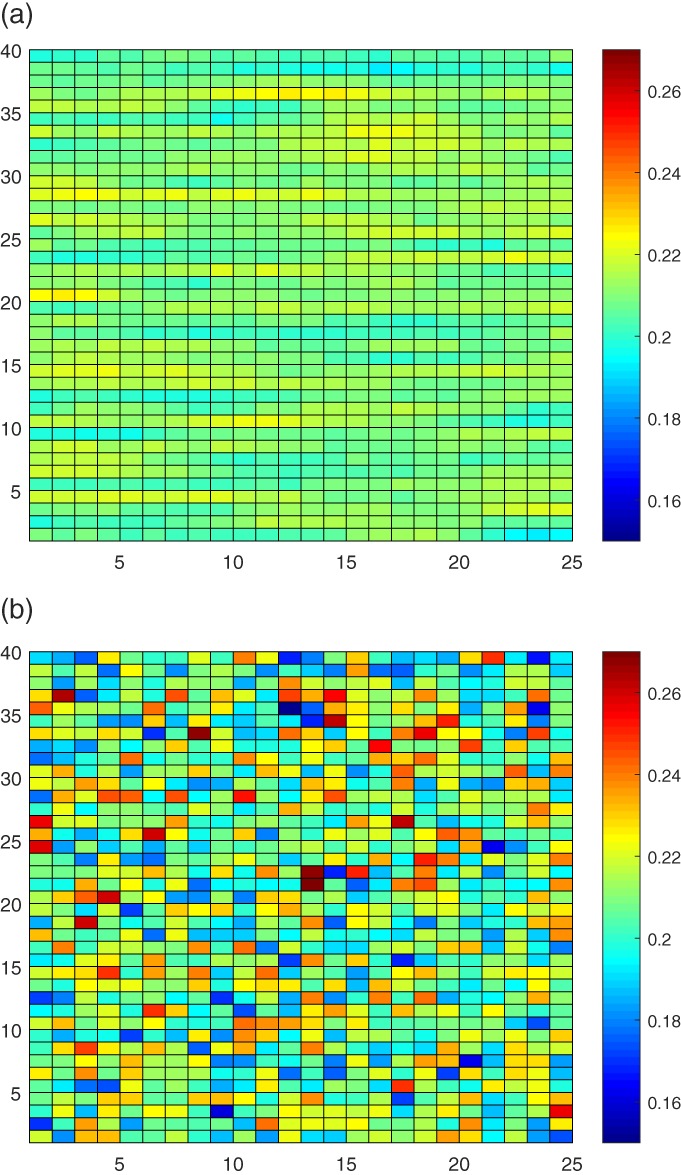
The main diagonal entries of estimated model error covariance **Q**_m_ with **R**=0.005, 40 ensemble members and **Q**0=0.25 (a) with and (b) without smoothing [Colour figure can be viewed at http://wileyonlinelibrary.com]

Figure 1 shows the 1000 main diagonal entries of the estimated model error covariance with and without smoothing after the 250 time steps, which are arranged in a 40×25 matrix in sequential order.

**Figure 2:**
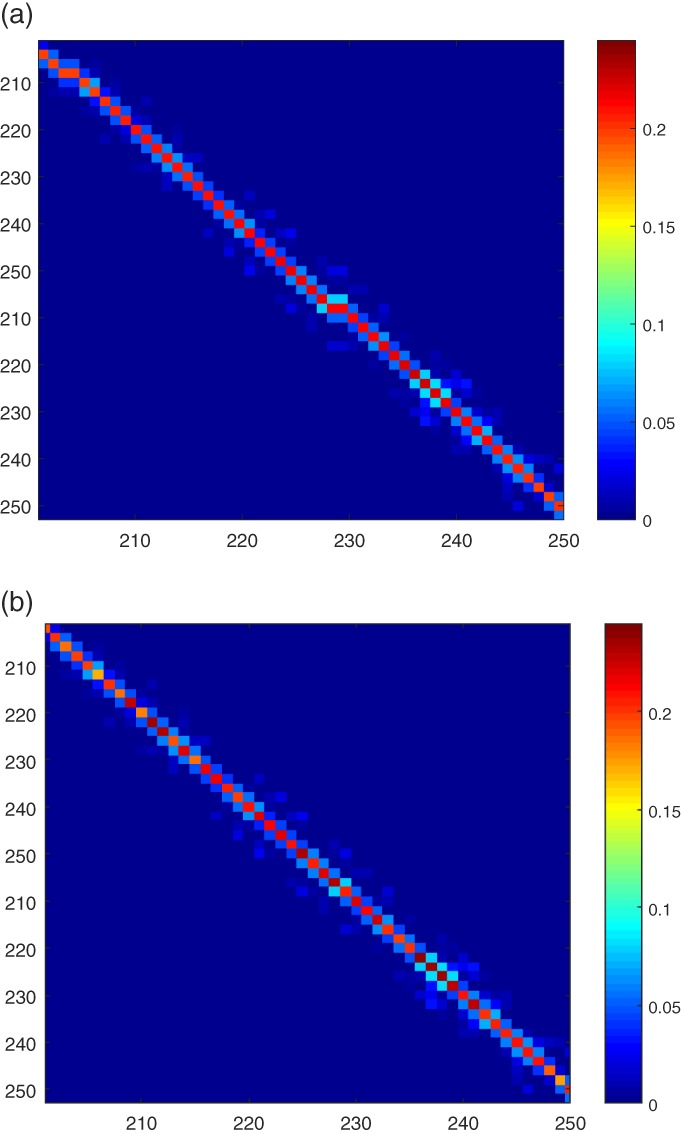
Enlarged part of the estimated model error covariance (a) with and (b) without smoothing [Colour figure can be viewed at http://wileyonlinelibrary.com]

It shows that smoothing strongly reduces the variance of the noisy main diagonal entries, leading to a more accurate covariance than without smoothing. Part of the full estimated model error covariance matrix with and without smoothing is given in Figure 2.

### 3.2 Sensitivity to ensemble size

Since the number of the ensemble members will always be much smaller than the rank of the model error covariance, we need to use the localization and smoothing described above. We investigate the impact of the ensemble size while incorporating this localization and smoothing. The ensemble size is increased from 40 to 200 and 400. The main diagonal value of observation error covariance is again set to 0.005. The initial first guess of model error covariance is again **Q**0=0.25**B**.

**Figure 3:**
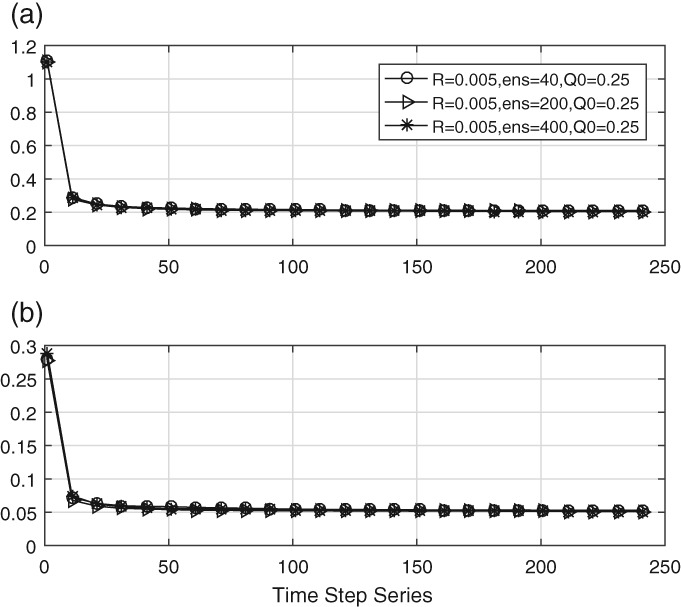
Time evolution of (a) the mean of the main diagonal entry and (b) the mean of the sub‐/super‐diagonal entries using three different ensemble sizes

Figure 3 depicts the time evolution of the estimated main and sub‐diagonals of the estimated **Q** matrix. What strikes the eye is the large value at initial times, much larger than the initial estimate of **Q**, which is 0.25 for the main diagonal. The reason is simply that the DA needs some time to adjust, and the values we see here are actually close to those of the initial background covariance matrix **B**. After a few time steps those values are forgotten and we settle quickly at the correct value, which is 0.2. This transient is visible in all experiments, both for the diagonal and the off‐diagonal elements.

The true values are 0.2 and 0.05. The figure shows that the ensemble size has little impact on the estimation methods. Therefore a small ensemble size of 40 is used in the rest of this paper. We do note, however, that this result does depend on the amount of localization and smoothing used in the estimate of **Q**.

### 3.3 Sensitivity to initial model error covariance Q**0**

In the following experiments we study the sensitivity of the estimation scheme to different initial covariance estimates **Q**0. Specifically, we test the behaviour of the scheme for **Q**0=0.5**B**, 0.25**B** and 0.1**B**. The observation error variance is set to be 0.005 and the ensemble size is 40.

**Figure 4:**
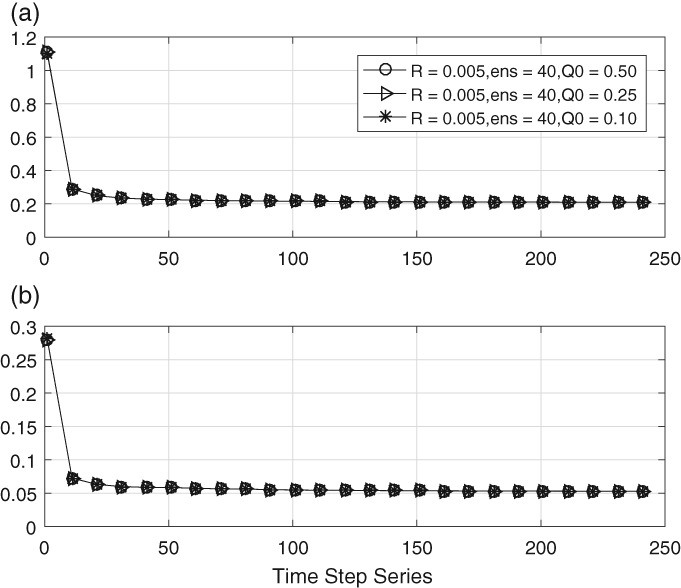
As Figure 3, but for three different values of Q**0**

Figure 4 illustrates that the convergence rate of the mean diagonal and super‐ and sub‐diagonal values is not sensitive to the value of **Q**0, at least not in the range tested here.

### 3.4 Sensitivity to observation error covariance R

In this section two studies will be performed. First we will test the sensitivity of the method to different values of the observation error covariance matrix **R**, followed by experiments in which we assume wrong values for **R** in the assimilation scheme.

We first test the behaviour of the scheme for diagonal values of the observation error covariance set to 0.005, 0.05, 0.1 and 0.2. Forty ensemble members are used in this experiment and the initial first guess is set to **Q**0=0.25**B**.

All values for **R** showed convergence of **Q** to the true value as above, except the largest value of **R**=0.2**I**. We found that when **R**=0.2**I** the estimation scheme is struggling to find good values for **Q** because the estimated matrix is not positive definite. Instead of fixing that directly, we used the practical approach which can easily be applied to larger systems too. Instead of using **Q**=**C**−**R**−2**V**, we use **Q**∼**C**−**R**−1.5**V** in our initial 150 estimates, and use **Q**=**C**−**R**−2**V** in the resulting 100 time steps.

**Figure 5:**
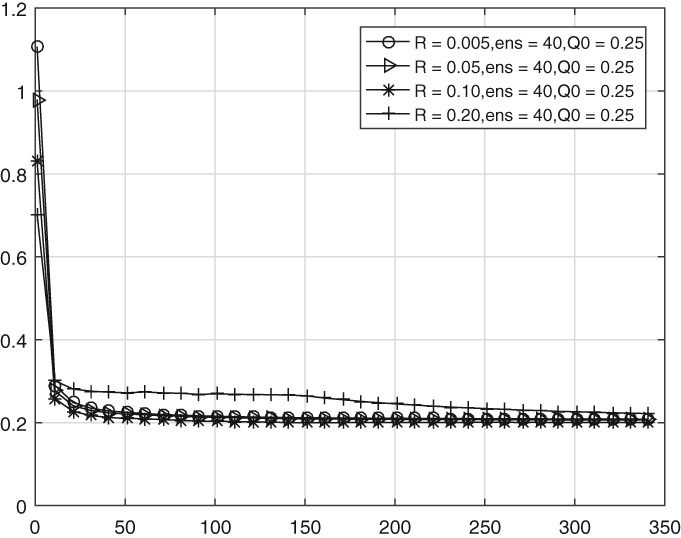
Time evolution over 350 time steps of the main diagonal entries of estimated model error covariance with four different values for **R**

**Figure 6:**
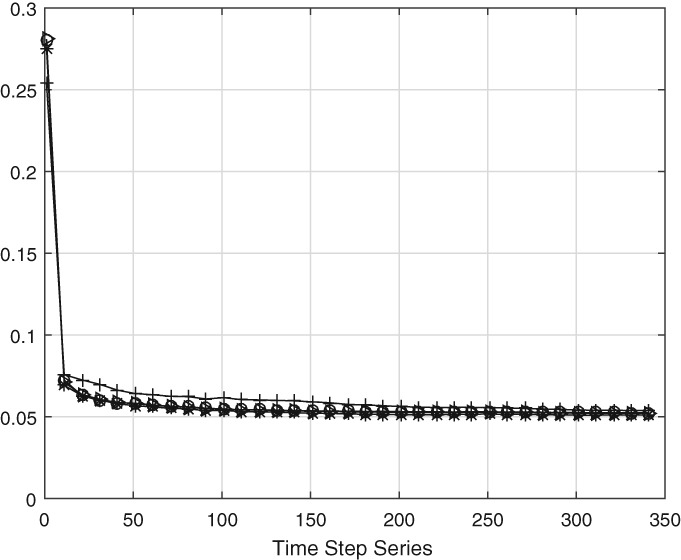
As Figure 5, but for the super‐ and sub‐diagonal entries of estimated model error covariance

Figures 5 and 6 show that accurate estimates for **Q** can be obtained, but the convergence is to a too high value of **Q** in the first 150 time steps. Using the correct equation for estimating **Q** in the rest of the time steps gives a slow but definite convergence to the true value. The slow convergence is thought to be due to two processes. Firstly, new estimates for **Q** contribute a small amount to the **Q** used in the DA scheme because the new estimate is averaged with all the old estimates of **Q**. Secondly, a larger value of elements in **R** will lead to a larger ensemble spread, so a larger amplitude of spurious fluctuations, so slower convergence to the actual value. With reference to Appendix A2, the slow convergence suggests that the matrices **A** ^*n*^ and **B** ^*n*^, which contain these ensemble averages, are larger here then for smaller **R**.

We now test the performance of the scheme when **R** is not known very accurately. This is the more realistic case. Our tests allow for a 50% error in **R**, so we test the scheme for diagonal values of **R** in the range 0.025, 0.05, and 0.075, with a true value of **R**=0.05.

**Figure 7:**
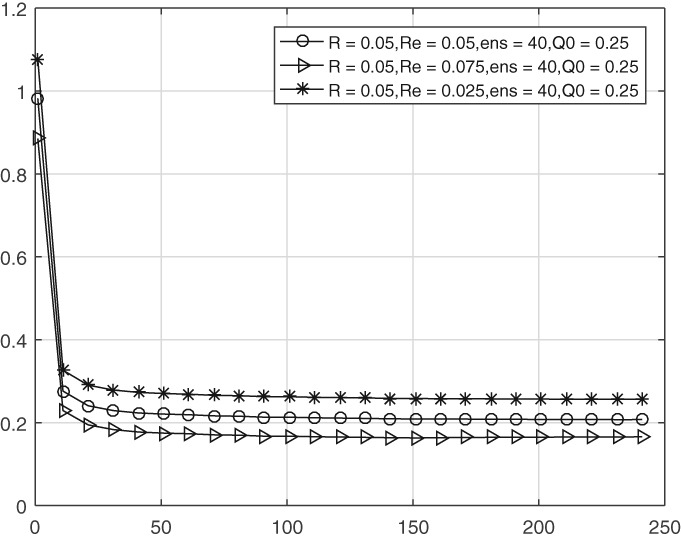
Time evolution of the main diagonal entries of estimated model error covariance with inaccurate **R**

**Figure 8:**
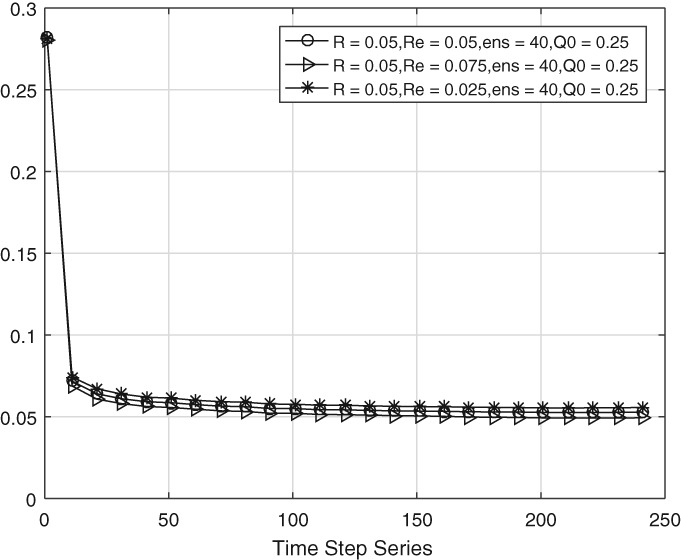
Time evolution of the sub‐/super‐diagonal entries of estimated model error covariance with inaccurate **R**

Figure 7 shows that the diagonal elements for **Q** are well estimated when the true **R** is used, but systematically too high and too low when the diagonal values of **R** are chosen too low and too high, respectively. This is a direct consequence of the way we calculate **Q**. On the other hand, as shown in Figure 8, the super‐ and sub‐diagonal elements in **Q** are affected to a much lesser extent, showing that only those elements in **Q** which are directly affected by **R** suffer most from an inaccurate **R** estimate.

## 4 CONCLUSIONS AND DISCUSSION

A new iterative scheme of estimating model error covariance in observation space, **HQH** ^*T*^, using particle filters is presented, exploring the innovation vectors of all particles. A simple temporal and spatial smoothing strategy is applied in the new scheme to speed up and stabilize the estimating process, increasing the rank of the estimated matrix and adding smoothness where we expect that to be appropriate. A convergence proof for the new method has also been developed.

The new scheme is easy to implement with the implicit equal‐weights particle filter. It was tested using numerical experiments on the 1000‐dimensional Lorenz (1996) model. The ensemble size has little impact on the estimating procedure, related to the temporal and spatial smoothing and localization applied. The resulting model error covariance matrices quickly converge to the correct matrix. These results are robust to varying initial guesses for **Q**, and varying sizes of observation error. Large values for the observation errors lead to slower convergence of the method as the ensemble spread will be larger, so the sampling errors are larger.

We also tested the method in the situation where **R** is not known accurately. The results are as reported for the Desroziers method when trying to estimate **H** **B** **H** ^*T*^, i.e. too low **R** values lead to too large **H** **B** **H** ^*T*^ values and vice versa (Desroziers *et al.*, 2005, 2009). Our numerical experiments indicate that only those elements of **Q** in which **R** is inaccurate are affected, but the generality of this statement is not tested, and is difficult to test exhaustively using only the simple model used here.

A weakness of the method is that we can only estimate **HQH** ^*T*^, so **Q** in observation space. Indeed, in the experiments we assumed that the whole state space is observed directly, which is not very realistic. For a full **Q** one will have to rely on spatial interpolation, with all problems related to that. Furthermore, **H** is assumed to be linear, or its linearization has to be accurate. However, apart from spatial smoothness we can explore the connection to the physics. Since **Q** is related to model physics, one could use that part of **Q** estimated in an observed area to infer **Q** in unobserved areas with similar physics. This is a clear advantage of estimating **Q** over **B**: **Q** is a matrix related to the model physics, while **B** is a matrix related to the accuracy of the state, which will vary over space and time, not directly related to the physics (Bannister, 2008a, 2008b).

We assumed that the **HQH** ^*T*^ matrix can be stored, which will not be true in realistic high‐dimensional applications. However, **Q**, and hence **HQH** ^*T*^ will be a narrow matrix, as it represents the influence of model errors which are largest at the model grid scale, with small horizontal length‐scales. So we only have to store the (block) diagonal and a few super‐ and sub‐ (block) diagonals, which should not be too problematic. This statement is not true for non‐local observations, such as several satellite observations that are line integrals. In that case **Q** is still sparse, but off‐diagonal elements can be large, and localization has to be done with care. Ideally one would like to store **Q** in operator form, but it is unclear at this moment how to estimate these operations directly. That would mean that one would need to generate **Q** ^1/2^ **x** for arbitrary **x**; this needs further research, which could be quite exciting in its own right.

The accuracy of the method for estimating **Q** is dependent on the accuracy which with we know **R**. Unfortunately, when our estimate of **R** is poor, we will get less accurate estimates for **Q** without knowing this to be the case; the method cannot detect issues like this. The most straightforward test might be to study the statistics of predictions, which is a non‐trivial exercise given the complex relation in a nonlinear model between errors in **Q** and errors in prediction statistics. In our experiments we only looked at diagonal **R**, while it is well known that **R** tends to be non‐diagonal, for instance due to representation errors (Stewart *et al.*, 2016; van Leeuwen, 2015b; Hodyss and Nichols, 2015). Although not studied, the expectation is that inaccurate off‐diagonal elements in **R** will lead to inaccurate non‐diagonal elements in **Q**. Another related issue is that we assumed that the observation operator **H** is known exactly, which is often not the case. To first order (Lorenc, 2011; van Leeuwen, 2015b), these errors can be incorporated in **R**, and the discussion above holds also for this case.

Although estimating **Q** is in its infancy, it is good to realise the importance of increasing the effort in this area. Estimated model errors give us a direct insight into which parts of the model are in error *at the level of the model equations*, allowing for direct model improvement. That would make DA into a tool much more powerful than is used at present. It is true that there are numerous issues with estimating **Q**, and no doubt we will never be able to estimate it completely, but that is not an argument for not trying. The potential benefits are enormous.

## APPENDIX A 1 ESTIMATING Q USING THE ANALYSIS ENSEMBLE

### 1.1

In this appendix we describe two other methods for estimating **HQH** ^*T*^, and show that they will be less efficient than the method presented in the main text.

#### A1 Estimating Q based on $(y^{n}\;-\;Hx_{i}^{n}){\left(y^{n}\;-\;Hx_{i}^{n}\right)}^{T}$

This method uses the analysis instead of the forecast to form the covariance, as:

(A1) $$C=\frac{1}{N_{e}-1}\sum_{i=1}^{N_{e}}(y^{n}-Hx_{i}^{n}){\left(y^{n}-Hx_{i}^{n}\right)}^{T}.$$

To evaluate this, we use, suppressing the time index *n*,

(A2) $$\begin{array}{ll} y-Hx_{i} & =y-Hx_{t}+Hx_{t}-Hx_{i}\;\\ & =y-Hx_{t}+Hx_{t}-Hf_{t}+Hf_{t}-Hx_{i}.\; \end{array}$$

Now we use the expression for **x**_*i*_ from the IEWPF, given by:

(A3) $$x_{i}=f_{i}+K(y^{n}-f_{i})+\alpha_{i}^{1/2}P^{1/2}\xi_{i}$$

leading to

(A4) $$\begin{array}{ll} y-Hx_{i} & =L(y-Hx_{t})+LH(x_{t}-f_{t})\;\\ & \;\;\;\;+LH(f_{t}-f_{i})-HP^{1/2}\alpha_{i}^{1/2}\xi_{i}, \end{array}$$

in which we defined **L** via

(A5) $$\begin{array}{ll} L & =(1-HK)\;\\ & =\{1-{HQH}^{T}{\left({HQH}^{T}+R\right)}^{-1}\}\;\\ & =R{\left({HQH}^{T}+R\right)}^{-1}.\; \end{array}$$

Using this expression in **C**, and assuming that all cross‐terms vanish, leads to

(A6) $$C\approx {LRL}^{T}+{LHQH}^{T}L^{T}+2{LVL}^{T}+W,$$

in which

(A7) $$V\approx \frac{1}{N-1}\sum_{i=1}^{N}(Hf_{i}-\bar{f}){\left(Hf_{i}-\bar{f}\right)}^{T}$$

and

(A8) $$W=HP^{1/2}\frac{1}{N-1}\sum_{i=1}^{N}\alpha_{i}\xi_{i}{\xi_{i}}^{T}{P^{1/2}}^{T}H^{T}.$$

We thus find

(A9) $${LHQH}^{T}L^{T}\approx C-L(R+2V)L^{T}-W,$$

which is similar to the expression for **HQH** ^*T*^ from the main text, but contains an extra matrix **L**, and **W**. To solve this equation, one can rewrite it as

(A10) $${LHQH}^{T}L^{T}=D$$

with

(A11) $$D=C-L(R+2V)L^{T}-W.$$

One way to solve this equation is to define **Z**=**HQH** ^*T*^ **L** ^*T*^ leading to

(A12) LZ=D,

and form an SVD of **L**=**U** *Λ* **V** ^*T*^ to find

(A13) $$Z=V\Lambda^{-1}U^{T}D.$$

Now solve **Z**=**HQH** ^*T*^ **L** ^*T*^ using the same SVD of **L** as

(A14) $${HQH}^{T}=V\Lambda^{-1}U^{T}DU\Lambda^{-1}V^{T}.$$

This method is much more involved as it needs the SVD of **L**, which can be prohibitively expensive when the dimension of the system is large. Other methods than the SVD could be explored, but that is beyond the scope of this paper.

#### A2 Estimating Q via $\{y^{n}\;-\;Hf(x_{i}^{n-1})\}{\left(y^{n}\;-\;Hx_{i}^{n}\right)}^{T}$

Finally, **Q** could be estimated exploring the cross‐covariance via

(A15) $$C=\frac{1}{N-1}\sum_{i=1}^{N}\left\{y^{n}-Hf\left(x_{i}^{n-1}\right)\right\}{\left(y^{n}-Hx_{i}^{n}\right)}^{T}.$$

To evaluate this, we again suppress the time index and use the expressions for (**y**−**H** *f*_*i*_) and (**y**−**H** **x**_*i*_) to find for **C**

(A16) $$C\approx {RL}^{T}+{HQH}^{T}L^{T}+2{VL}^{T},$$

in which

(A17) $$\begin{array}{ll} & V\approx \frac{1}{N-1}\sum_{i=1}^{N}(Hf_{i}-H\bar{f}){\left(Hf_{i}-H\bar{f}\right)}^{T}\; \end{array}$$

and

(A18) L=(1−HK).

Furthermore, we assumed that the *α*_*i*_ term cancels with the other terms on average.

We can rewrite this equation as

(A19) $${HQH}^{T}L^{T}=D,$$

with

(A20) $$D=C-(R+2V)L^{T}.$$

Define the SVD of **L** as **L**=**U** *Λ* **V** ^**T**^ to find

(A21) $${HQH}^{T}=DU\Lambda^{-1}V^{T}.$$

This method is better conditioned than the method above, but still needs the SVD of **L**.

## APPENDIX B 1 CONVERGENCE OF THE ESTIMATION METHOD

### 1.1

First recall that the update equation for the IEWPF from section 2.3.1 is

(B1) $$x_{i}^{n}=f(x_{i}^{n-1})+K^{n}(y^{n}-Hf_{i}^{n})+{\left(P^{n}\right)}^{1/2}{\left(\alpha_{i}^{n}\right)}^{1/2}\xi_{i}^{n}$$

in which now

(B2) $$f_{i}^{n}=f(x_{i}^{n-1}),$$

(B3) $$K^{n}=Q^{n}H^{T}{\left({HQ}^{n}H^{T}+R\right)}^{-1},$$

and

(B4) $$P^{n}={\left\{{\left(Q^{n}\right)}^{-1}+H^{T}R^{-1}H\right\}}^{-1},$$

and **Q** ^*n*^ is our current estimate of **Q**.

In our estimate of **C** and **V**, we need to calculate $f_{i}^{n}$ which can be written as:

(B5) $$\begin{array}{ll} f_{i}^{n} & =f(x_{i}^{n-1})\;\\ & =f\{f_{i}^{n-1}+K^{n-1}(y^{n-1}-Hf_{i}^{n-1})\;\\ & \;\;+{\alpha_{i}^{n-1}}^{1/2}{P^{n-1}}^{1/2}\xi_{i}^{n-1}\}.\; \end{array}$$

For ease of notation, we drop the time index from now on, noting that **Q**=**Q** ^*n*−1^, **P**=**P** ^*n*−1^, **K**=**K** ^*n*−1^, *α*=*α* ^*n*−1^, and $\xi_{i}=\xi_{i}^{n-1}$. Let us now write our estimate **Q** in terms of the true covariance **Q**_*t*_ and a systematic perturbation, as

(B6) $$Q=Q_{t}+ɛ,$$

in which we assume that the matrix *ɛ* is much smaller in magnitude than **Q**_*t*_. Also note that *ɛ*=*ɛ* ^*n*−1^, so it is a time‐dependent matrix. Then we can write, to first order in *ɛ*,

(B7) $$\begin{array}{ll} K & =(Q_{t}+ɛ)H^{T}{\left\{H(Q_{t}+ɛ)H^{T}+R\right\}}^{-1}\;\\ & \approx K_{t}+ɛH^{T}{\left({HQ}_{t}H^{T}+R\right)}^{-1}\;\\ & \;\;\;\;-K_{t}HɛH^{T}{\left({HQ}_{t}H^{T}+R\right)}^{-1}\;\\ & =K_{t}+(1-K_{t}H)ɛH^{T}{\left({HQ}_{t}H^{T}+R\right)}^{-1}.\; \end{array}$$

For matrix **P** we find:

(B8) $$\begin{array}{ll} P & ={\left\{{\left(Q_{t}+ɛ\right)}^{-1}+H^{T}R^{-1}H\right\}}^{-1}\;\\ & =Q-{QH}^{T}{\left({HQH}^{T}+R\right)}^{-1}HQ\;\\ & =Q_{t}+ɛ-(Q_{t}+ɛ)H^{T}{\left\{H(Q_{t}+ɛ)H^{T}+R\right\}}^{-1}(Q_{t}+ɛ)\;\\ & \approx Q_{t}-Q_{t}H^{T}{\left({HQ}_{t}H^{T}+R\right)}^{-1}{HQ}_{t}+(1-K_{t}H)ɛ\;\\ & \;\;\;-(1-K_{t}H)ɛH^{T}K_{t}^{T}\;\\ & =P_{t}+(1-K_{t}H)ɛ{\left(1-K_{t}H\right)}^{T}\;\\ & =P_{t}+P_{t}Q_{t}^{-1}ɛQ_{t}^{-1}P_{t}.\; \end{array}$$

We need the square root of **P** ^*n*^ in the direction of *ɛ*. A first‐order Taylor series expansion for matrix functions reads, for *μ* a small scalar,

(B9) $$g(X+\mu Y)\approx g(X)+\mu {\left.\frac{d}{d\mu }\right|}_{\mu =0}g(X+\mu Y).$$

So we write *ɛ*=*μ* **E**, in which *μ* is a small scalar, and use *g*(*X*)=*X* ^1/2^ in the above equation to find:

(B10) $$\begin{array}{ll} P^{1/2} & ={\left(P_{t}+\mu P_{t}Q_{t}^{-1}{EQ}_{t}^{-1}P_{t}\right)}^{1/2}\;\\ & \approx P_{t}^{1/2}+\mu {\left.\frac{d}{dt}\right|}_{\mu =0}{\left(P_{t}+\mu P_{t}Q_{t}^{-1}{EQ}_{t}^{-1}P_{t}\right)}^{1/2}\;\\ & =P_{t}^{1/2}+\frac{1}{2}\mu P_{t}^{-1/2}P_{t}Q_{t}^{-1}{EQ}_{t}^{-1}P_{t}\;\\ & =P_{t}^{1/2}+\frac{1}{2}P_{t}^{1/2}Q_{t}^{-1}ɛ\;Q_{t}^{-1}P_{t}\;.\; \end{array}$$

**Q** also appears in *α*_*i*_, but that dependence is rather complicated as the argument of an exponential that is part of a complicated argument of the Lambert W function. Instead we will argue below that we do not need to explore this further.

Using these first‐order approximations, we find for $f(x_{i}^{n})$, to first order in *ɛ*,

(B11) $$\begin{array}{ll} & f_{i}^{n}=f(x_{i}^{n-1})\;\\ & =f\left\{f_{i}^{n-1}+K_{t}(y^{n-1}-Hf_{i}^{n-1})+{{\alpha_{i}}_{t}^{n-1}}^{1/2}{P_{t}}^{1/2}\xi_{i}^{n-1}\right\}\;\\ & \;+F_{i}^{n}(1-K_{t}H)ɛH^{T}{\left({HQ}_{t}H^{T}+R\right)}^{-1}(y^{n-1}-Hf_{i}^{n-1})\;\\ & \;-\frac{1}{2}F_{i}^{n}{{\alpha_{i}}_{t}}^{1/2}P_{t}^{1/2}Q_{t}^{-1}ɛ\;Q_{t}^{-1}P_{t}\xi_{i}\;\\ & \;+F_{i}^{n}{\left(\frac{\partial {{\alpha_{i}}_{t}}^{1/2}}{\partial Q_{t}}\right)}^{T}ɛ\;P_{t}^{1/2}\xi_{i}, \end{array}$$

in which $F_{i}^{n}$ is the linearized model at state $x_{i}^{n-1}$.

We now develop **C** to first order in *ɛ*, leading to

(B12) $$\begin{array}{ll} & C^{n}=C_{t}^{n}+\frac{1}{N-1}\sum_{i=1}^{N}(y^{n}-Hf_{i}^{n}){\left(y^{n-1}-Hf_{i}^{n-1}\right)}^{T}\;\\ & \;\;\;\times {\left({HQ}_{t}H^{T}+R\right)}^{-1}Hɛ{\left(1-K_{t}H\right)}^{T}F^{T}H^{T}+{\left(..\right)}^{T}\;\\ & -\frac{1}{2}\frac{1}{N-1}\sum_{i=1}^{N}(y^{n}-Hf_{i}^{n}){\xi_{i}^{T}\alpha_{i}}_{t}^{1/2}P_{t}Q_{t}^{-1}ɛQ_{t}^{-1}P_{t}^{1/2}F^{T}H^{T}\;\\ & \;\;\;\;\;\;+{\left(..\right)}^{T}\;\\ & +\frac{1}{N-1}\sum_{i=1}^{N}(y^{n}-Hf_{i}^{n}){\xi_{i}}^{T}P_{t}^{1/2}ɛ\frac{\partial {\alpha_{i}}_{t}}{\partial Q_{t}}F^{T}H^{T}+{\left(..\right)}^{T}, \end{array}$$

where (..)^*T*^ denotes the transpose of the previous term in the equation. First we note that, since *ξ*_*i*_ is independent from all other factors and has mean zero when averaged over the ensemble, so all terms that contain *ξ*_*i*_ will be small, and even zero for an infinite ensemble size. We cannot use that argument for the second term and its transpose because the $\left(y^{n}\;-\;Hf_{i}^{n}\right)$, $\left(y^{n-1}\;-\;Hf_{i}^{n-1}\right)$ and $F_{i}^{n}$ have non‐zero ensemble means. In fact, using the same development as in Eq. (2), we find that the ensemble mean for these terms is ${ɛ}^{n}\;+\;\beta_{t}^{n}$, ${ɛ}^{n-1}\;+\;\beta_{t}^{n-1}$, and ${\bar{F_{i}^{n}}}^{T}=F^{T}$ respectively, for large ensemble sizes. However, these terms are dependent over time, so when we average over time in the first part of the scheme used to estimate **Q**, these terms become small also, and this is further amplified by smoothing over space.

For the sample matrix **V** ^*n*^ we find

(B13) $$\begin{array}{ll} V^{n}= & V_{t}^{n}+\frac{1}{N-1}\sum_{i}(H\bar{f^{n}}-Hf_{i}^{n}){\left(H\bar{f^{n-1}}-Hf_{i}^{n-1}\right)}^{T}\;\\ & \;\;\times {\left({HQ}_{t}H^{T}+R\right)}^{-1}Hɛ{\left(1-K_{t}H\right)}^{T}F^{T}H^{T}+{\left(..\right)}^{T}\;\\ & -\frac{1}{2}\frac{1}{N-1}\sum_{i=1}^{N}(H\bar{f^{n}}-Hf_{i}^{n})\xi_{i}^{T}{{\alpha_{i}}_{t}}^{1/2}\;\\ & \;\;\;\times P_{t}Q_{t}^{-1}ɛ\;Q_{t}^{-1}P_{t}^{1/2}F^{T}H^{T}+{\left(..\right)}^{T}\;\\ & +\frac{1}{N-1}\sum_{i=1}^{N}(H\bar{f^{n}}-Hf_{i}^{n})\xi_{i}^{T}P_{t}^{1/2}ɛ\frac{\partial {\alpha_{i}}_{t}}{\partial Q_{t}}F^{T}H^{T}+{\left(..\right)}^{T}, \end{array}$$

and a similar convergence argument applies as for the RHS terms of **C** ^*n*^.

In the second part of the iterative scheme, we use the estimated **Q** in the DA. The update scheme is

(B14) $$\begin{array}{ll} Q^{n} & =C^{n}-R-2V^{n}\;\\ & =C_{t}-R-2V_{t}+A^{n}{ɛ}^{n-1}{\left(B^{n}\right)}^{T}+B^{n}{ɛ}^{n-1}{\left(A^{n}\right)}^{T}\;\\ & =Q_{t}+A^{n}{ɛ}^{n-1}{\left(B^{n}\right)}^{T}+B^{n}{ɛ}^{n-1}{\left(A^{n}\right)}^{T}, \end{array}$$

in which **A** ^*n*^ and **B** ^*n*^ are small and depend only weakly on *ɛ* ^*n*−1^, as argued above. Furthermore, we can write

(B15) $$Q^{n}=Q_{t}+{ɛ}^{n}\;.$$

Combining these two equations, we find

(B16) $$Q^{n}=Q_{t}+A^{n}{ɛ}^{n-1}{B^{n}}^{T}+B^{n}{ɛ}^{n-1}{A^{n}}^{T}=Q_{t}+{ɛ}^{n}.$$

We thus find for *ɛ* ^*n*^ the recursion

(B17) $${ɛ}^{n}=A^{n}{ɛ}^{n-1}{B^{n}}^{T}+B^{n}{ɛ}^{n-1}{A^{n}}^{T},$$

and we can write

(B18) $$|{ɛ}^{n}|\leq 2|A^{n}|\;|{ɛ}^{n-1}|\;|B^{n}|\;.$$

Since |**A** ^*n*^| and |**B** ^*n*^| are small as argued above, this iteration will make *ɛ* ^*n*^ smaller and smaller with increasing *n*, so that **Q** ^*n*^ converges to **Q**_*t*_. The numerical experiments confirm this convergence on a nonlinear 1000‐dimensional problem.
